# Supplementary figures and images for: Transcriptome Dynamics of Pseudomonas aeruginosa during Transition from Overlapping To Non-Overlapping Cell Cycles
Source: mSystems. 2023 Feb 14;8(2):e01130-22. doi: 10.1128/msystems.01130-22 (PMC10134858; doi:10.1128/msystems.01130-22)

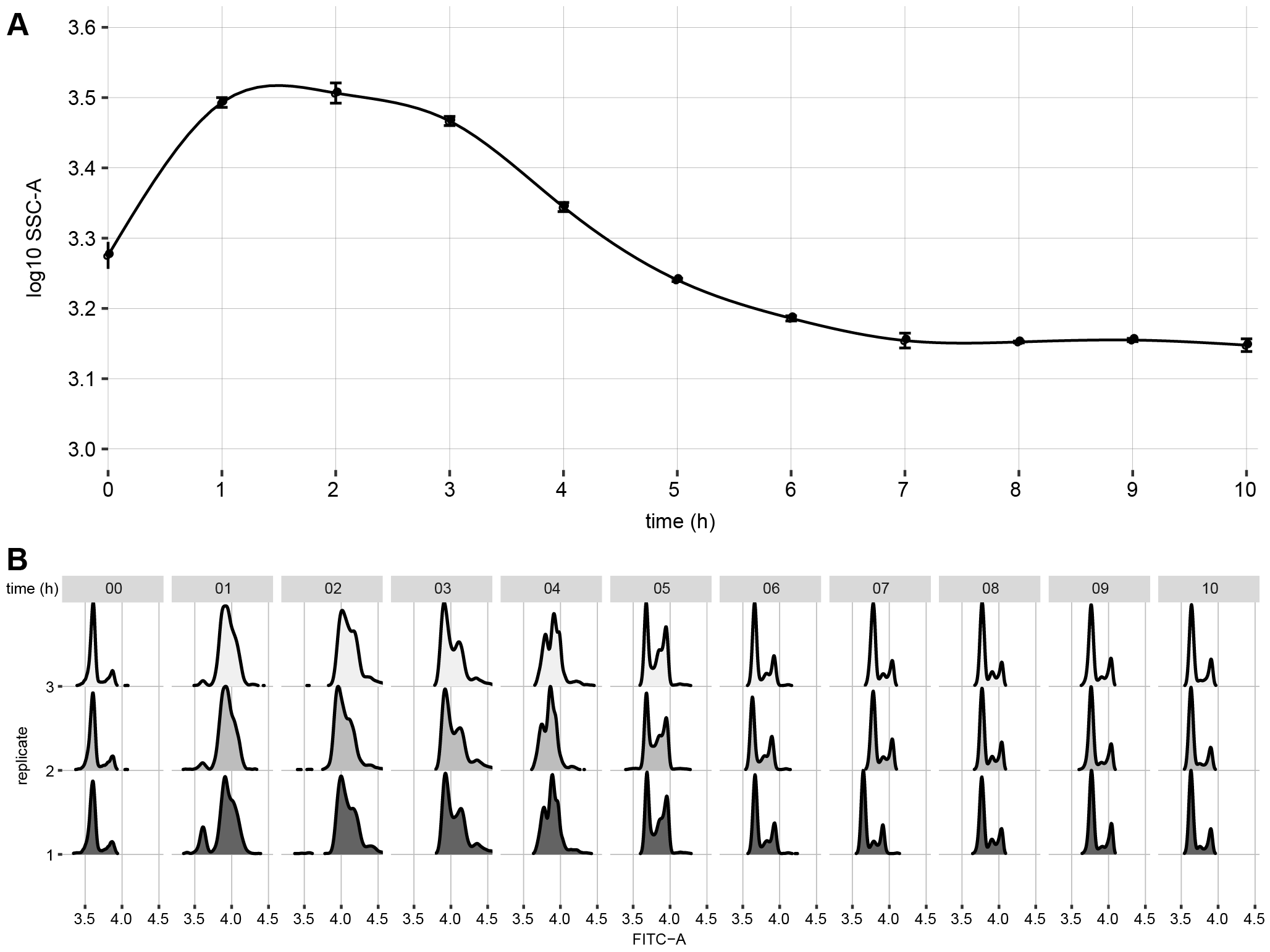

Supplement: FIG S1 [file msystems.01130-22-s0001.tif]

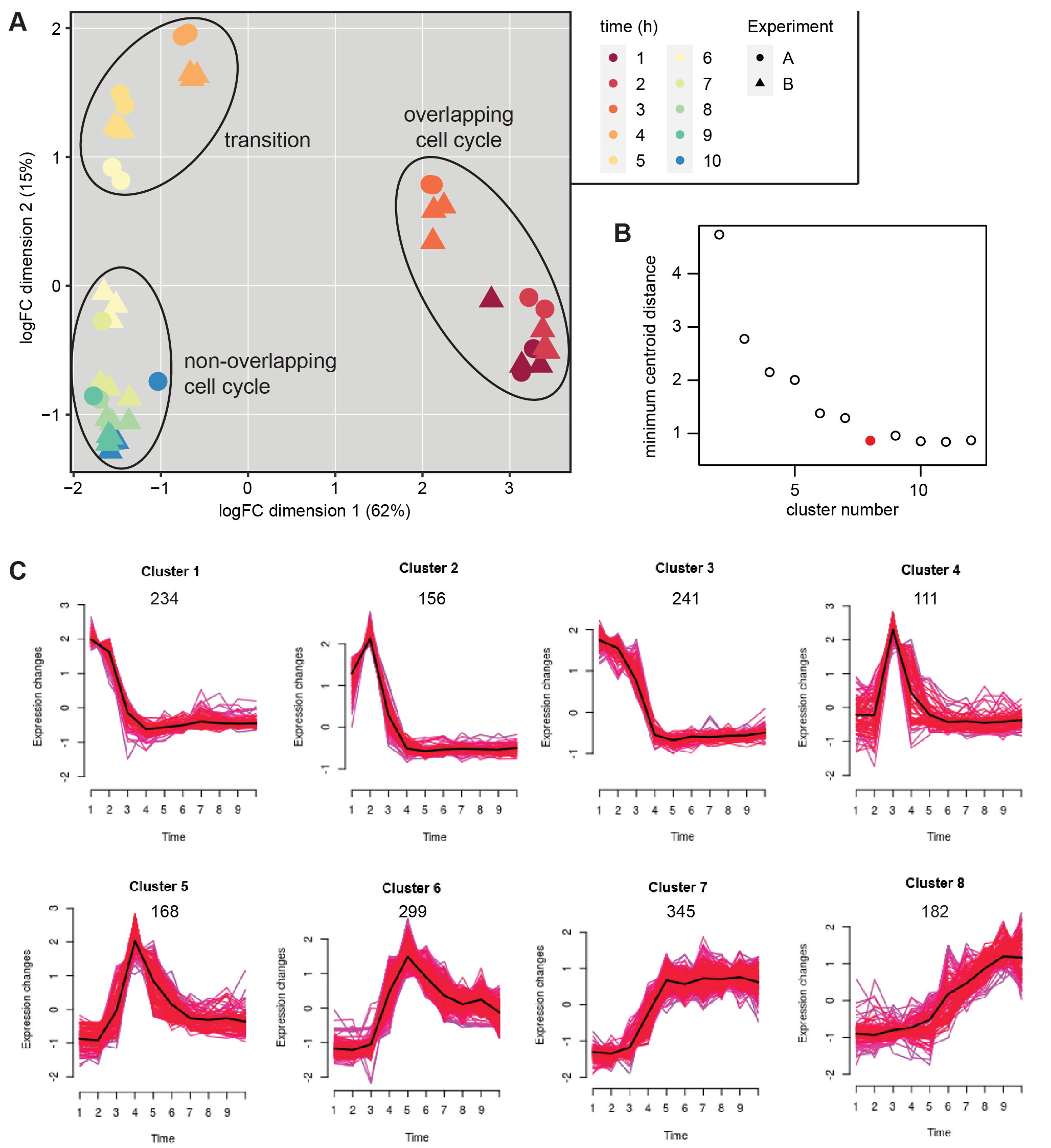

Supplement: FIG S2 [file msystems.01130-22-s0002.tif]

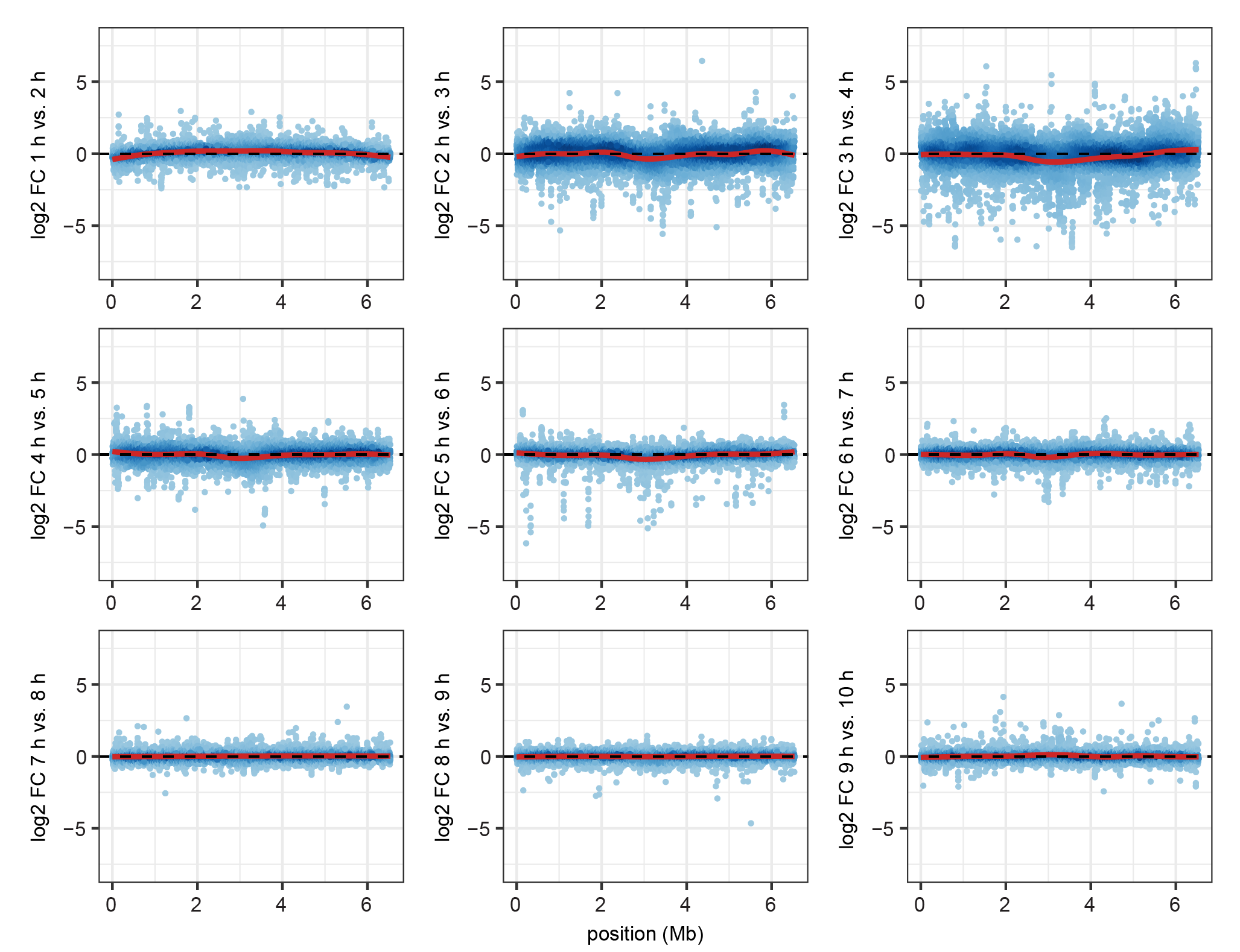

Supplement: FIG S3 [file msystems.01130-22-s0003.tif]

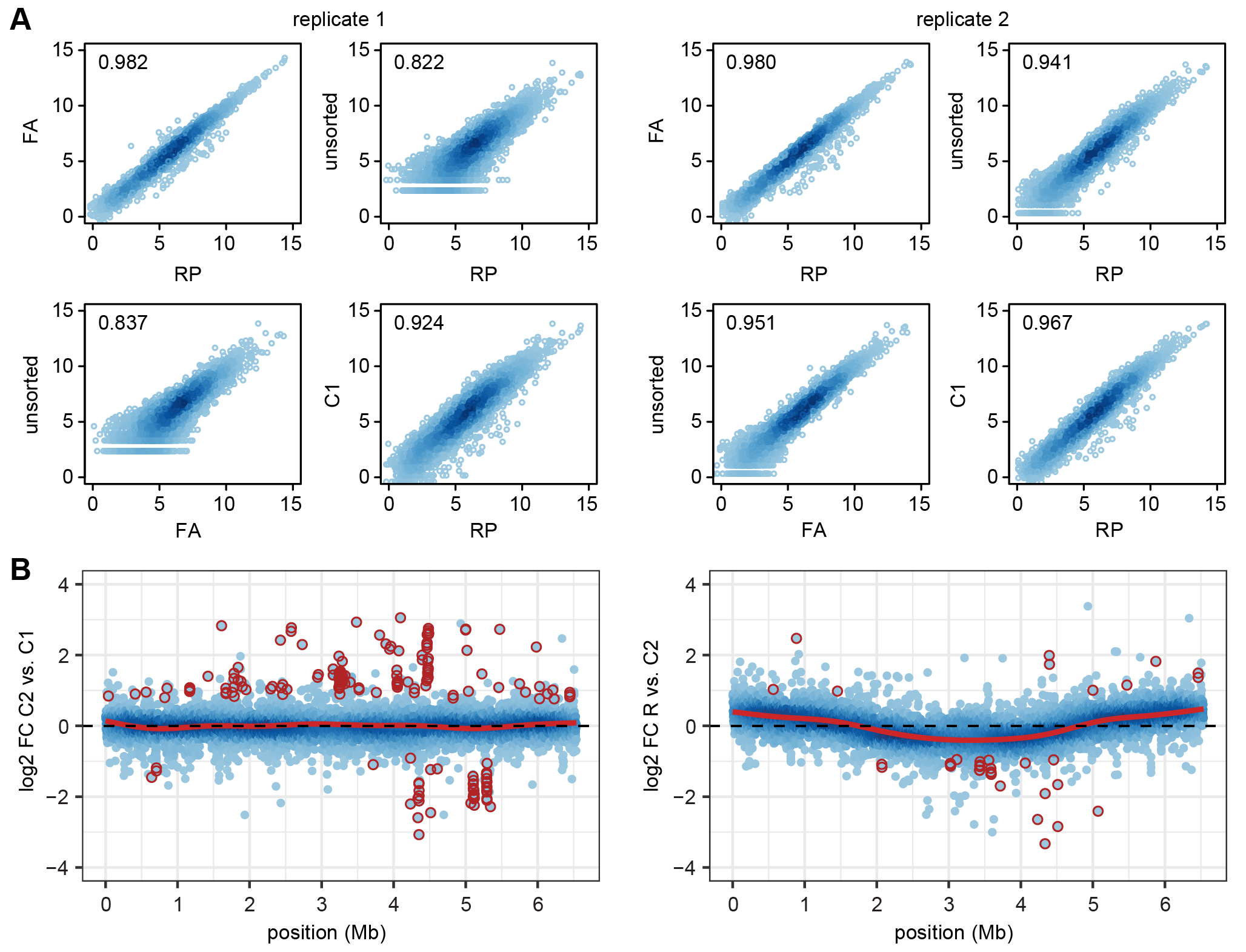

Supplement: FIG S4 [file msystems.01130-22-s0004.tif]
